# Evaluation of Genetic Diversity in Sugar Beet Using SCoT and ISSR Markers

**DOI:** 10.3390/plants15040613

**Published:** 2026-02-14

**Authors:** Betül Yücel, Yeter Çilesiz, Tolga Karaköy

**Affiliations:** 1 Graduate School of Education, Sivas University of Science and Technology, Sivas 58140, Türkiye; 2 Department of Field Crops, Faculty of Agricultural Sciences and Technology, Sivas University of Science and Technology, Sivas 58140, Türkiye; ycilesiz@sivas.edu.tr; 3 Department of Plant Protection, Faculty of Agricultural Sciences and Technologies, Sivas University of Science and Technology, Sivas 58140, Türkiye; tkarakoy@sivas.edu.tr

**Keywords:** SCoT, ISSR, variation, sugar beet, genetic diversity

## Abstract

Sugar beet (*Beta vulgaris* L.) is an economically important crop that accounts for approximately 20% of global sugar production. The success of future breeding programs depends on the effective utilization of existing genetic resources. The aim of this study was to assess the genetic diversity and population structure of 192 sugar beet (*Beta vulgaris* L.) genotypes, including commercial cultivars and accessions obtained from the USDA gene bank, using SCoT and ISSR molecular markers, and to identify potential genetic resources for sugar beet breeding programs. In this study, a total of 192 sugar beet genotypes, including 187 accessions from the USDA (U.S. Department of Agriculture) gene bank and 5 commercial cultivars, were evaluated for genetic diversity using Start Codon Targeted (SCoT) and Inter Simple Sequence Repeat (ISSR) markers. A total of 68 scorable bands were obtained from five SCoT and three ISSR primers, and all bands were found to be polymorphic (100% polymorphism). Parameters such as polymorphic information content (PIC), Nei’s genetic diversity, and Shannon’s index indicated a high level of variation within the gene pool, with SCoT markers being more informative than ISSR markers. Dendrogram analyses based on Nei’s genetic distance revealed that the populations were separated into two main groups, while the sub-clusterings contained broad genetic variation. STRUCTURE analysis identified four (K = 4) populations for the SCoT data and three (K = 3) populations for the ISSR data; the inclusion of a high number of individuals in the admixture population indicated extensive gene flow. Principal component analysis (PCA) revealed both homogeneous groups and differentiated genotypes contributing to within-population diversity. The results demonstrate that the combined use of SCoT and ISSR markers provides powerful and complementary tools for assessing genetic diversity in sugar beet. The findings provide a solid scientific basis for the development of new, high-yielding and high-quality sugar beet cultivars as well as for the conservation of existing genetic resources. Molecular data constitute an important reference for guiding sugar beet breeding programs and for the effective utilization of genetic resources.

## 1. Introduction

Sugar beet (*Beta vulgaris* L.) is an important industrial crop belonging to the Amaranthaceae family [1,2]. Sugar beet is a biennial and cross-pollinated plant with a diploid genome structure (2n = 18) [3] and accounts for approximately 20% of global sugar production [4]. In addition to being used as an important raw material for carbohydrate supply, biofuel, and pharmaceutical industries, its by-products, pulp and molasses, are widely used in animal nutrition [5,6]. Sugar beet cultivation, which has spread across Europe and other regions of the world, has today formed a broad genetic resource encompassing both commercial cultivars and their wild relatives [7,8]. However, the narrow genetic base of modern cultivars poses a serious threat to breeding programs, particularly under changing climatic conditions [9,10]. Molecular marker systems are widely used in sugar beet for assessing genetic diversity, constructing high-resolution genetic maps, and identifying quantitative trait loci (QTLs) associated with agronomically important traits [11,12,13]. Success in breeding programs largely depends on genetic diversity that provides new alleles for traits such as yield, stress tolerance, and disease resistance. Among DNA-based markers, RAPD, ISSR, SSR, and AFLP have been widely used in genetic diversity studies in sugar beet [14,15,16,17,18,19]. Among these markers, start codon targeted (SCoT) markers stand out as an effective tool for evaluating genetic diversity [20,21]. SCoT markers are derived from conserved regions flanking the ATG start codon of plant genes and are classified as dominant markers with high reproducibility [22]. Because they do not require prior sequence information and are low-cost, they can also be used in species with limited genomic resources. SCoT markers have been successfully applied for various purposes in many economically important crops, including genetic diversity analysis, cultivar and hybrid identification, construction of linkage maps, and genetic fidelity analysis of tissue culture-derived plants. The use of SCoT and ISSR markers in screening sugar beet germplasm will provide important information on population structure and genetic diversity and will contribute to the effective utilization of these resources in breeding programs. The primary rationale for using SCoT and ISSR markers in combination in this study was to enable the assessment of genetic diversity in sugar beet at different genomic levels through a complementary approach. Although RAPD, ISSR, SSR, and AFLP markers have been widely used in genetic diversity studies of sugar beet, each marker system targets distinct regions of the genome and therefore reflects different types of biological information. SCoT markers target gene-associated (functional) regions surrounding the ATG start codon, allowing the detection of genetic variation that may be associated with selection and breeding processes. In contrast, ISSR markers amplify regions between microsatellite loci and thus primarily represent neutral, genome-wide variation. Consequently, relying solely on SCoT markers could have limited the assessment of genetic diversity to functional regions only. In this context, the combined use of SCoT and ISSR markers enabled the comparative analysis of variation in gene-associated and neutral genomic regions, the evaluation of the consistency of genetic patterns obtained from different marker systems, and a more comprehensive and reliable interpretation of genetic structure relevant to sugar beet breeding programs [6,7,10,23].

In this study, the genetic diversity of 186 genotypes obtained from the USDA gene bank and five commercial cultivars was evaluated using SCoT and ISSR markers, and the genetic relationships within sugar beet germplasm were comprehensively elucidated.

## 2. Materials and Methods

### 2.1. Plant Materials

In this study, 187 sugar beet genotypes from the USDA Agricultural Research Service (USDA) genebank, along with 5 commercial beet varieties (Serenada, Varias, Evelina, Jaguar, Balaban), were used for genetic characterization (Table 1).

### 2.2. DNA Isolation

The 192 samples were grown under field conditions at the Agricultural R&D Center of Sivas University of Science and Technology. During the early emergence stage of the plants, approximately 100 mg of fresh leaf tissue was collected and transferred to the laboratory. DNA isolation was performed with minor modifications to the cetyltrimethylammonium bromide (CTAB) method described by Doyle and Doyle (1990) [24]. The leaves were frozen in liquid nitrogen and ground into a fine powder using a porcelain mortar and pestle. A buffer composed of extraction buffer, lysis buffer, and sarcosyl solutions was heated to 65 °C and then added to the powdered samples. The samples were incubated at 65 °C for 30 min, followed by the addition of chloroform–isoamyl alcohol (24:1) and mixing. After centrifugation at 6000× *g* for 20 min at 21 °C, the upper phase was transferred to a new tube and cold (−20 °C) isopropanol was added. Following centrifugation at 6000× *g* for 5 min at 21 °C, the supernatant was discarded and washing buffer (70% EtOH) was added. After incubation at room temperature for 20 min, the samples were centrifuged at 6000× *g* for 10 min at 21 °C and the supernatant was removed. The pellet was washed with cold ethanol and, after centrifugation at 6000× *g* for 10 min at 21 °C, the upper phase was discarded and the pellet was dissolved in 100 μL of ultrapure water. Stock DNA concentrations were measured using a MaestroNano Pro spectrophotometer (MN913A, MaestroGen, Hsinchu, Taiwan), and DNA samples were diluted to a final concentration of 5 ng/µL.

### 2.3. SCoT Marker Assay

A preliminary screening was performed using 36 SCoT primers defined by Collard and Mackill on ten different sugar beet genotypes [22]. The aim was to identify primers producing clear and well-defined polymorphic banding profiles. As a result of the preliminary screening, five SCoT primers (SCoT-1, SCoT-4, SCoT-15, SCoT-28, and SCoT-32) that produced the best polymorphic bands were selected for screening all genotypes (Table 2). For the evaluation of SCoT polymorphism, all isolated DNA samples were screened using these five markers developed by Collard and Mackill [22]. The PCR reaction mixture consisted of 4 μL DNA (20 ng), 1 μL primer, 10 μL PCR master mix (Eco Tech, Cat. No: ET5), and 10 μL dH_2_O. A total of 20 ng of template DNA was used in a 25 μL PCR reaction. The PCR conditions included an initial denaturation for 3 min, followed by 35 cycles of denaturation at 94 °C for 1 min, annealing at 54–61 °C for 1 min, and extension at 72 °C for 1 min, with a final extension at 72 °C for 10 min. For electrophoresis of PCR products, a 2% agarose gel prepared in Tris–borate–EDTA buffer was used. The gel was stained with ethidium bromide and visualized using a UV imaging system (Bio-Rad Laboratories, Inc., Hercules, CA, USA).

### 2.4. ISSR Marker Assay

A preliminary screening was conducted using 18 ISSR primers on ten different sugar beet genotypes. The aim was to identify primers producing clear and well-defined polymorphic banding profiles. As a result of the preliminary screening, three ISSR primers (UBC-831, UBC-836, and UBC-840) that produced the best polymorphic bands were selected for screening all genotypes (Table 2). For the evaluation of ISSR polymorphism, all isolated DNA samples were screened using these three markers. The PCR reaction mixture consisted of 4 μL DNA (20 ng), 1 μL primer, 10 μL PCR master mix (Eco Tech, Cat. No: ET5), and 10 μL dH_2_O. A total of 20 ng of template DNA was used in a 25 μL PCR reaction. The PCR conditions included an initial denaturation for 3 min, followed by 35 cycles of denaturation at 94 °C for 1 min, annealing at 50–52 °C for 1 min, and extension at 72 °C for 1 min, with a final extension at 72 °C for 10 min. For electrophoresis of PCR products, a 2% agarose gel prepared in Tris–borate–EDTA buffer was used. The gel was stained with ethidium bromide and visualized using a UV imaging system (Bio-Rad Laboratories, Inc., Hercules, CA, USA).

### 2.5. Statistical Analysis

During scoring, the presence of a band was coded as 1 and the absence of a band as 0. Evaluations were focused only on bright, clear, and well-resolved bands [25]. Diversity parameters such as the effective number of alleles [26], Nei’s (1973) gene diversity [27], and Shannon’s Information Index were calculated using POPGENE v.1.32 software [28]. The average polymorphic information content (PIC) for each SCoT and ISSR primer was calculated using the following formula (Equation (1)) [29]:

PIC = 2fi (1 − fi)(1)

where PIC represents the polymorphic information content, fi is the frequency of band presence, and 1 − fi indicates band absence. The Nei’s genetic distance matrix obtained from POPGENE was used to construct a neighbour-joining dendrogram using MEGA software. The population structure of sugar beet genotypes was investigated using STRUCTURE software. The optimal number of clusters (K subpopulations) was estimated by performing the analyses three times for K values ranging from 1 to 10 [30,31]. In each run, the burn-in period and Markov chain Monte Carlo (MCMC) length were set to 50,000, and the number of iterations was set to 10. The resulting outputs were then processed using STRUCTURE HARVESTER v.0.9.94 software to determine the optimal K value [32]. To evaluate genetic relationships among sugar beet populations, Principal Component Analysis (PCA) was performed using MVSP 3.22 [33].

**Table 2:** Primers used for SCoT and ISSR assay, their sequence, GC content, Tm value, number of bands and diversity parameters.

Markers		Sequence (5′-3′)	GC%	Tm °C	Number of Bands		Diversity Parameter				
					Polymorphic Bands	Total Bands	P%	ne	h	I	PIC
**SCoT**	SCoT-1	CAACAATGGCTACCACCA	50	54	12	12	100	1.83	0.44	0.63	0.44
	SCoT-4	CAACAATGGCTACCACCT	50	54	6	6	100	1.76	0.41	0.60	0.41
	SCoT-15	ACGACATGGCGACCGCGA	67	61	15	15	100	1.42	0.27	0.44	0.27
	SCoT-28	CCATGGCTACCACCGCCA	67	61	10	10	100	1.54	0.32	0.48	0.32
	SCoT-32	CCATGGCTACCACCGCAC	67	61	8	8	100	1.50	0.31	0.47	0.31
**ISSR**	UBC-831	CTCTCTCTCTCTCTCTT	47	50	6	6	100	1.73	0.41	0.59	0.41
	UBC-836	AGAGAGAGAGAGAGAGYA	44	52	5	5	100	1.47	0.28	0.44	0.29
	UBC-840	GAGAGAGAGAGAGAGAAT	44	52	6	6	100	1.69	0.39	0.58	0.39

ne: Effective number of alleles, h: Nei’s (1973) gene diversity [27], I: Shannon’s Information index, PIC: polymorphism information contents.

## 3. Results and Discussion

### 3.1. Diversity Shown Through SCoT and ISSR Assay

A total of eight primers that produced strong PCR results were selected for genotyping all sugar beet samples (Table 2). Using these primers, a total of 68 scorable bands were obtained. A total of 51 scorable bands were obtained from the SCoT primers. All of these bands were polymorphic, with an average of 8.5 bands per primer. A total of 17 scorable bands were obtained from the ISSR primers. The lower number of bands produced by ISSR primers compared to SCoT primers in this study can be attributed to structural differences in the genomic regions targeted by the two marker systems. ISSR markers amplify the regions between microsatellite repeats (SSR regions) in the genome, the distribution and frequency of which may vary depending on the genome and species. In sugar beet, the relatively limited distribution and lower polymorphism of ISSR target regions may have resulted in a reduced number of amplified bands. In contrast, SCoT markers are designed based on the conserved start codon (ATG) regions of genes targeting gene-rich and more conserved genomic regions, which facilitates the generation of a higher number of reproducible and scorable bands. The number of bands per primer ranged from 5 (UBC-836) to 15 (SCoT-15). The use of these primers enabled the detection of a high level of polymorphism among the 192 samples. The polymorphism rate observed in these samples was calculated as 100%.

According to Table 2, among all primers, SCoT-1 exhibited the highest effective number of alleles (1.83), while SCoT-15 showed the lowest value (1.42). Gene diversity determined by SCoT primers ranged from 0.27 (SCoT-15) to 0.44 (SCoT-1), whereas gene diversity estimated using ISSR primers ranged from 0.28 (UBC-836) to 0.41 (UBC-831). The Shannon diversity index varied between 0.44 (SCoT-15) and 0.63 (SCoT-1) for SCoT primers, and between 0.44 (UBC-836) and 0.59 (UBC-831) for ISSR primers. Regarding PIC values, SCoT-1 had the highest value (0.44) and SCoT-15 had the lowest value (0.27) among all primers (Table 2). The higher polymorphism rates and PIC values observed for SCoT markers compared to ISSR markers can be explained by the structural and functional differences in the genomic regions targeted by these two marker systems. SCoT markers are gene-targeted markers that amplify conserved yet functionally variable regions flanking the ATG start codon of genes, which may be more susceptible to both natural and artificial selection pressures [22,23]. In particular, selective processes acting on genes associated with stress tolerance, environmental adaptation, and yield may contribute to increased allelic diversity in these regions [10]. In contrast, ISSR markers are primarily based on the amplification of regions between microsatellites located in non-coding and relatively neutral parts of the genome, and are therefore less influenced by selective forces [18]. Consequently, the higher genetic diversity detected by SCoT markers may reflect not only technical differences between marker systems but also the accumulation of evolutionary- and breeding-related variation in functionally relevant genomic regions [23]. This indicates that SCoT and ISSR markers are complementary, and that their combined use enables a more comprehensive assessment of both functional and neutral genetic variation [18].

When the two different primer groups used in this study were compared based on the results presented in Table 2, both primer groups were found to provide high levels of polymorphism. However, SCoT primers were evaluated as more powerful and informative than ISSR primers for the assessment of genetic diversity, as they produced a higher number of bands and yielded higher diversity indices (PIC, h, I).

Wild and cultivated beets were clearly distinguished using RFLP markers, and it was reported that wild beets possessed broader genetic variation than cultivated forms, with fodder beets in particular exhibiting low genetic diversity [34]. In sugar beet, very high polymorphism rates (93–97.2%) and high genetic diversity indices (0.86–0.91) were detected using RAPD and ISSR markers, demonstrating that both marker systems were effective in revealing differences among genotypes [35]. It was also reported that RAPD and ISSR markers captured a wider range of genetic variation compared to isozymes and provided high discriminatory power [36]. In studies employing SSR markers, a substantial proportion of genetic variation was found to occur among populations (48%), and clear genetic distances among lines were observed [37]. Furthermore, SSR markers were shown to offer strong resolution for discriminating sugar beet genotypes, with high PIC values ranging from 0.78 to 0.91 [38]. In a more recent study, polymorphism rates exceeding 97% and moderate PIC values were reported using SCoT markers [39]. Taken together, these studies consistently demonstrate that findings obtained using different molecular marker systems reliably reveal the level of genetic diversity and inter-population differentiation in sugar beet and that the results are supportive of the findings of the present study.

### 3.2. Genetic Distance

**Figure 1:**
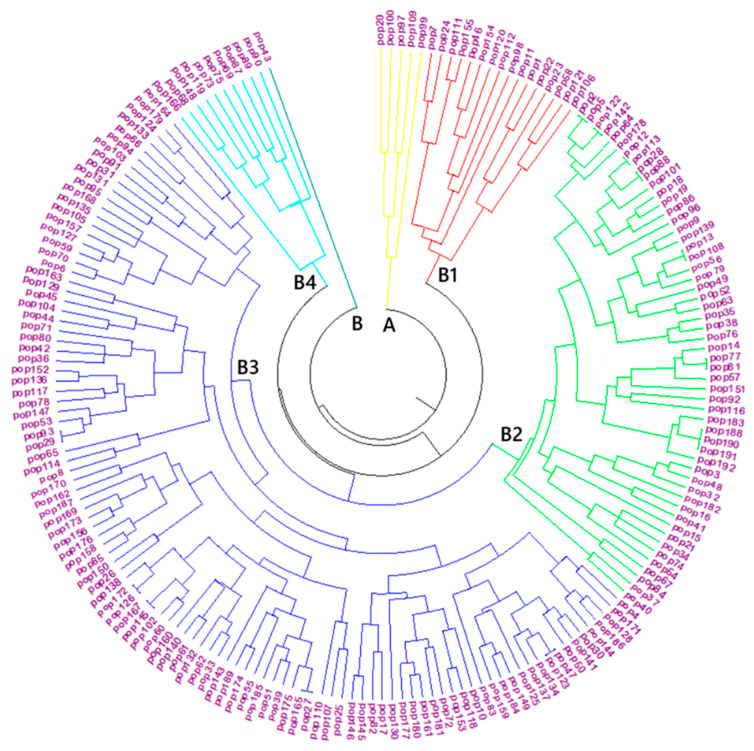
Clustering of sugar beet germplasm based on a neighbour-joining dendrogram constructed using Nei’s genetic distance (A (yellow), B (dark blue), B1 (red), B2 (green), B3 (blue), B4 (light blue)).

Based on the circular neighbor-joining dendrogram constructed using Nei’s genetic distance, the genetic structure of the analyzed populations was separated into two main groups (A and B) (Figure 1). Group A consisted of a limited number of samples and exhibited a compact structure that was clearly separated from all other groups in the dendrogram. This indicates that, although Group A populations possess a narrow genetic diversity, they represent a distinct gene pool. Therefore, this group has critical potential for the conservation and utilization of alternative genetic resources in breeding programs.

Group B, on the other hand, was divided into four subclusters (B1, B2, B3, and B4), revealing the presence of broader genetic variation. The samples in cluster B1 showed a high level of similarity to each other and, with their homogeneous structure, likely represent materials sharing a common selection history. In contrast, cluster B2, which contained a large number of populations and exhibited extensive branching diversity, emerged as the subgroup with the widest genetic base. This group is considered a rich source of variation that may harbor diverse traits and can be exploited in breeding programs. Clusters B3 and B4 displayed relatively more homogeneous structures but were clearly differentiated from each other. Populations in B3 are important for variety development due to their genetic proximity, whereas populations in B4 likely harbor different genetic variants associated with environmental adaptation. These findings clearly reveal not only the levels of genetic diversity within the populations but also their subpopulation structure.

Overall, the resulting dendrogram demonstrates that the molecular markers used accurately reflect the relationships among populations and provide a guiding dataset for both the conservation of genetic resources and breeding efforts. Using SCoT markers, very high levels of polymorphism (92.85%) and high PIC values (0.783–0.907) were obtained in sugarcane [40]. Based on UPGMA analysis, 132 sugar beet cultivars were grouped into six main genetic clusters [41]. Similar average Nei’s genetic distances (≈0.24) were obtained using DAMD and SCoT markers [11]. Using the UPGMA method, 106 sugar beet germplasm accessions were separated into four main groups [12]. Finally, high PIC values (0.58–0.83) and distinct clustering patterns were achieved using iPBS markers [42]. Taken together, these studies indicate that different PCR-based markers such as SCoT, RAPD, DAMD, and iPBS are effective in revealing the level of genetic diversity and population structure in sugar beet and related species and that the clustering patterns obtained strongly support the findings of the present study.

### 3.3. Population Structure Based on STRUCTURE Analysis

**Figure 2:**
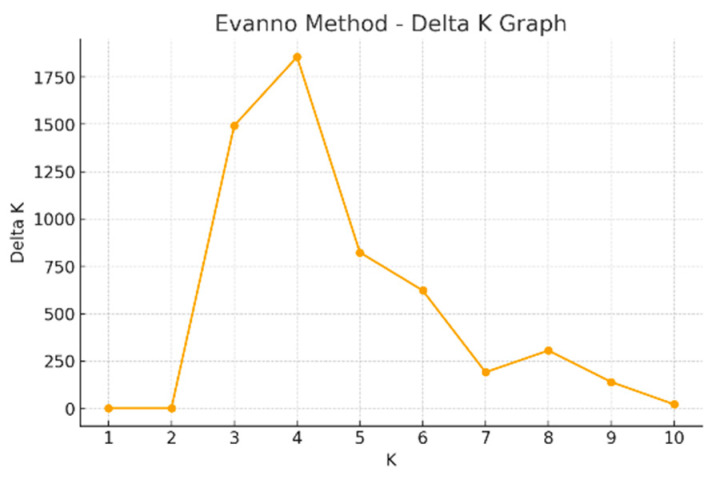
The K value of the sugar beet population indicates the presence of four populations when analyzed using SCoT primers.

In the analysis of population structure, the number of populations (K) was evaluated over a range from 1 to 10 (SCoT). According to the ΔK criterion proposed by Evanno et al. (2005) [31], the highest ΔK value was observed at K = 4 (Figure 2). This result corresponds to the presence of four populations among the analyzed individuals.

**Figure 3:**
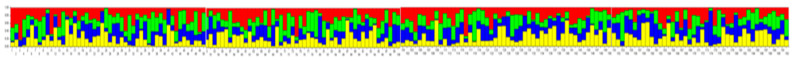
Population structure of the sugar beet population inferred at K = 4 (SCoT). Population A (red) includes 6 samples, Population B (green) 14 samples, Population C (blue) 6 samples, Population D (yellow) 9 samples, while the admixture population consists of 157 samples (membership coefficient ≥ 0.75).

In the bar plot obtained from the STRUCTURE analysis (Figure 3), the membership probabilities of each individual to different gene pools are represented by colors. Based on the membership coefficient criterion of ≥0.75, the majority of individuals were assigned to four main populations; however, a certain proportion could not be clearly assigned to any single population. These individuals were therefore classified as an admixture population. These results indicate that the analyzed populations show a substantial level of genetic differentiation and that the genetic material can be grouped into four main clusters. Furthermore, the presence of admixture individuals suggests ongoing gene flow among populations, which represents an important factor shaping the genetic structure.

**Figure 4:**
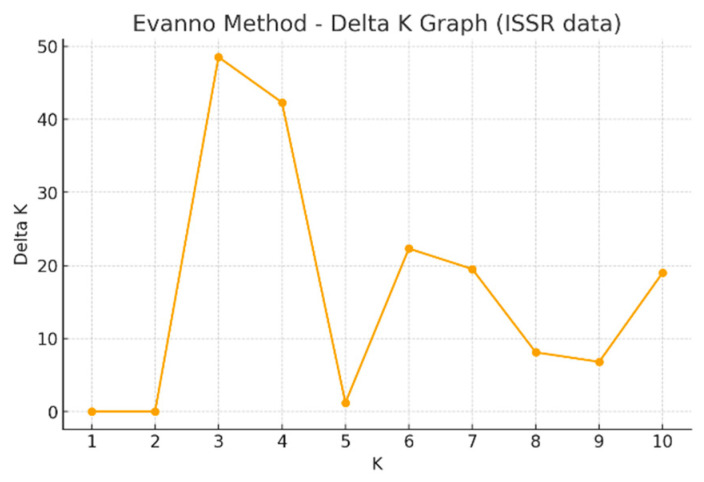
The K value of the sugar beet population indicates the presence of three populations when analyzed using ISSR primers.

In the analysis of population structure, the number of populations (K) was evaluated over a range from 1 to 10 (ISSR). According to the ΔK criterion proposed by Evanno et al. (2005), [31] the highest ΔK value was observed at K = 3 (Figure 4). This result indicates the presence of three main gene pools among the analyzed individuals.

**Figure 5:**
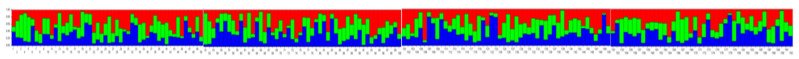
Population structure of the sugar beet population inferred at K = 3 (ISSR). Population A (red) includes 35 samples, Population B (green) includes 43 samples, Population C (blue) 98 samples, while the admixture population consists of 16 samples (membership coefficient ≥ 0.75).

In the bar plot obtained from the STRUCTURE analysis (Figure 5), the membership probabilities of each individual to different gene pools are represented by colors. Based on the membership coefficient criterion of ≥0.75, the majority of individuals were assigned to three main populations; however, a certain proportion could not be clearly assigned to any single population.

Population structure in the *Beta vulgaris* complex was investigated using Bayesian clustering analysis, and individuals were assigned to two main clusters (K = 2) [43]. Using SSR and InDel markers, population structure analysis of monogerm sugar beet germplasm identified K = 2 as the most appropriate number of populations [44]. STRUCTURE analysis of elite sugar beet genotypes also yielded the highest value at K = 2 [45]. In contrast, population structure was best explained by three genetic groups (K = 3) [46]. Taken together, these studies indicate that Bayesian-based population structure analyses in sugar beet and the *Beta vulgaris* complex generally point to two main genetic groups; however, depending on the scope of the material and the extent of genetic diversity, higher K values may also emerge, which is consistent with the STRUCTURE results obtained in the present study.

**Figure 6:**
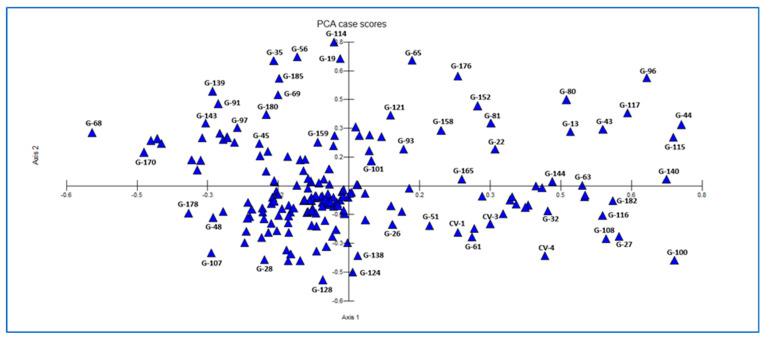
PCA Plot of 192 Sugar Beet Genotypes Based on SCoT Markers.

Figure 6 presents the two-dimensional PCA distribution, in which the first two components (Axis 1 and Axis 2) explain a substantial proportion of the genetic variation among genotypes. The majority of genotypes (G-1, G-26, G-28, G-45, G-51, G-61) are clustered near the center of the coordinate plane, indicating that they possess highly similar genetic structures, whereas several genotypes located at the extremes (G-68, G-170, G-178, G-96, G-140, G-100) exhibit high genetic distances and contribute most strongly to within-population diversity. In addition, the separation of genotypes, such as G-114, G-65, and G-19 along the positive direction of the second component and genotypes such as G-124, G-128, and G-138 along the negative direction of the second component, indicates partial differentiation within the population. The control cultivars (CV-1, CV-3, CV-4) are positioned close to the central region, suggesting that their genetic structures are similar to those of the broader population and that they are suitable representatives of overall diversity.

**Figure 7:**
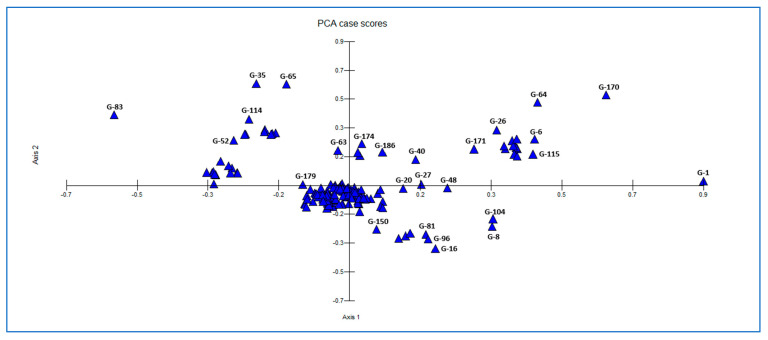
PCA Plot of 192 Sugar Beet Genotypes Based on ISSR Markers.

In the two-dimensional PCA distribution shown in Figure 7, the first two components account for a substantial proportion of the genetic variation among genotypes. The majority of genotypes are densely clustered near the center of the coordinate plane (G-20, G-27, G-40, G-48, G-150, G-179), indicating a high degree of genetic similarity among them. In contrast, genotypes located at the extremes (G-1, G-83, G-170) are clearly separated from the center and stand out as individuals contributing most strongly to within-population diversity. In addition, genotypes such as G-35, G-52, G-65, and G-114 are differentiated along the positive direction of the second component, whereas genotypes such as G-8, G-16, G-81, G-96, and G-104 are separated along the negative direction of the second component, forming small subgroups. This distribution indicates that the population is generally homogeneous, while certain individuals represent distinct gene pools due to their greater genetic distances.

Genetic similarity among exotic sugar beet cultivars was shown to be largely shaped by breeding companies; however, the presence of cultivars from different companies within the same clusters was attributed to the narrow genetic base of sugar beet and the exchange of genetic resources among breeding programs [41]. In a large-scale genomic study, wild beet populations belonging to the genus Beta were divided into two distinct subgroups of Mediterranean and Atlantic origin based on PCA, phylogenetic, and admixture analyses, with higher levels of genetic differentiation observed in Atlantic populations [47]. Using SRAP markers, high levels of polymorphism, wide similarity ranges (0.58–0.93), and five major genetic clusters were identified in chard genotypes [48]. In multigerm sugar beet germplasm, four major genetic groups were identified, and most of the genetic variation was shown to originate from within-population differences (93%) [12]. Employing SSR and InDel markers, monogerm sugar beet lines were divided into two main populations and several subgroups, with high genetic distances observed between specific genotype pairs, highlighting their potential importance as parental lines in breeding programs [44]. Similarly, moderate to high polymorphism (67.11%), wide genetic similarity ranges (0.485–0.925), and a generally homogeneous yet broadly variable distribution in PCA analysis were reported for 18 sugar beet genotypes evaluated using SCoT markers [49]. Taken together, these studies indicate that different marker systems, including genomic, SSR, SRAP, and SCoT markers, provide complementary and consistent insights into the level of genetic diversity, population structure, and identification of breeding-relevant genotypes within the genus Beta and sugar beet [50,51,52].

## 4. Conclusions

This study demonstrates that the combined use of SCoT and ISSR markers is highly effective for assessing genetic diversity and population structure in sugar beet germplasm. The 100% polymorphism observed across all primers indicates that the evaluated material possesses broad genetic variation. Clustering and STRUCTURE analyses revealed clear population stratification, while the high proportion of admixed individuals suggests strong gene flow among populations. The higher diversity indices obtained with SCoT markers compared to ISSR markers indicate that SCoT markers are more powerful in resolving genetic relationships. The identification of genetically differentiated individuals suggests that these genotypes may serve as valuable sources of alleles related to stress tolerance, disease resistance, and yield improvement. Overall, these findings emphasize the importance of conserving sugar beet genetic resources and effectively utilizing them in breeding programs, providing a strong foundation for sustainable production and cultivar development. The genetic diversity and population structure analyses presented in this study are not limited solely to the identification of basic genetic resources but also provide a framework for identifying materials that can be directly utilized in parent selection for sugar beet breeding programs. The genetic characterization of genotypes that were monitored under field conditions for four years and subjected to preliminary selection based on phenotypic observations using molecular markers enables the identification of genotypes possessing desired agronomic and adaptation traits as genetically distinct and complementary parents. This approach makes a significant contribution to the expansion of the genetic base in breeding programs, the development of combinations with targeted traits, and the implementation of more rational parent selection in hybridization studies. Therefore, this study provides a concrete resource for future breeding efforts focused on yield, quality, stress tolerance, and disease resistance by establishing a genotypic and phenotypic-based parent pool.

## Figures and Tables

**Table 1:** Details of sugar beet populations used in the study.

Genotype No	Plant Name	Registration Number	Genotype No	Plant Name	Registration Number
1	Ames 8283	B151	97	PI 120694	No. 1714
2	Ames 8286	B169	98	PI 590684	C22
3	PI 169017	Pancar	99	PI 142815	CHOGHONDAR
4	PI 193458 Ames 15638	No. 8525	100	Ames 3060	IDBBNR 4825
5	Ames 15638	BO 100	101	PI 163176	PALOG
6	Ames 8287	B176	102	Ames 8292	B192
7	Ames 8295	B199	103	PI 120692	No. 1539
8	PI 596528	Rs-2b	104	NSL 6346	LUCULLUS
9	NSL 28024	Extra Early	105	PI 610417	EL40 BREEDING LINE 6 and 12
10	PI 105335	TZU LO PU TOU	106	PI 590581	US 015
11	PI 611062	B55650	107	PI 590808	MS EQUIVALENT OF CT 9
12	Ames 8284	B159	108	NSL 176412	YUGO 7
13	PI 142814	CHOGHONDAR	109	PI 140355	No. 6624
14	Ames 8302	B0152	110	PI 610291	A77-46
15	PI 140354	No. 6526	111	PI 113306	No. 323
16	PI 169032	No. 3709	112	PI 164671	No. 9096
17	PI 590621	Extra Early Red	113	Ames 8447	Thurles I
18	PI 164968	No. 44	114	PI 148625	CHAGHONDA
19	Ames 4436	IDBBNR 5767	115	PI 171508	No. 6728
20	PI 171518	No. 7164	116	PI 169014	No. 1394
21	PI 611059	Ticha	117	Ames 8281	B139
22	PI 179176	No. 9892	118	PI 120705	No. 3208
23	Ames 4377	IDBBNR 4836	119	PI 140361	No. 7178
24	PI 613264	GW 035	120	PI 179845	PALAK
25	Ames 3096	IDBBNR 4828	121	PI 174060	No. 8296
26	PI 608800	A78-32	122	NSL 188575	NS-358 (C1)
27	PI 176875	No. 9335	123	NSL 6319	KING RED
28	Ames 4265	IDBBNR 5652	124	NSL 86579	72/4-41-2-T4
29	Ames 4331	IDBBNR 4831	125	Ames 2632	L 4T
30	PI 120691	No. 1379	126	Ames 10838	10603
31	PI 124528	CHAKUNDA	127	Ames 2652	SLC 35
32	PI 142809	CHOGHONDAR	128	NSL 176410	YUGO 5
33	Ames 4375	IDBBNR 4834	129	NSL 31344	GIANT YELLOW ECKENDORF
34	PI 142812	CHOGHONDAR	130	NSL 142007	044
35	Ames 3039	IDBBNR 4811	131	Ames 2631	L 3T
36	Ames 8288	B180	132	PI 176425	No. 8972
37	Ames 8297	B229	133	Ames 2656	SLC 128
38	Ames 2661	SLC 132	134	PI 109040	No. T-169
39	PI 144675	No. 8148	135	PI 610317	1564AA
40	NSL 28716	WYOMING NO 09	136	PI 590584	US 035-0
41	PI 140357	No. 6820	137	PI 169028	No. 2960
42	PI 140360	No. 7121	138	PI 171513	No. 6883
43	Ames 4219	IDBBNR 5606	139	PI 590582	US 056/2
44	PI 142810	CHOGHONDAR	140	NSL 95223	A77-52
45	Ames 3047	IDBBNR 4819	141	NSL 28026	GARDENERS MODEL
46	Ames 8448	Thurles 2	142	PI 175597	KOCABAS
47	PI 120707	No. 3264	143	Ames 15637	BO-85
48	PI 142816	CHOGHONDAR	144	PI 169019	No. 1844
49	PI 610286	A76-36	145	NSL 28719	WYOMING NO 18
50	PI 172733	No. 7647	146	PI 109039	No. T-184
51	PI 176427	KOCABAS	147	NSL 141985	JANASZ
52	PI 610323	C301CMS	148	PI 176873	KOCABAS
53	PI 140356	No. 6627	149	NSL 43404	PARMA GLOBE
54	PI 169029	PANCAR	150	NSL 93285	A77-17
55	PI 142811	CHOGHONDAR	151	NSL 28714	WYOMING NO 04
56	PI 142808	No. 7352	152	PI 613230	GW 389
57	PI 165485	CHOGHUNDAR	153	PI 590593	IMPROVED EARLY EGYPTIAN
58	Ames 8293	B195	154	PI 590607	EARLY BLOOD TURNIP
59	Ames 10841	WB 765	155	PI 590683	C04
60	Ames 8291	B189	156	PI 610328	750-2
61	PI 164659	No. 9084	157	PI 610266	US 022/4
62	Ames 4376	IDBBNR 4835	158	Ames 2634	L 8T
63	Ames 2658	SLC 131	159	Ames 2659	SLC 131
64	PI 179180	CICLA	160	PI 590580	US 033
65	PI 610287	A76-38	161	PI 590704	WB 7
66	PI 120695	No. 1814	162	PI 613270	A 0051
67	PI 120693	No. 1656	163	NSL 6320	WINTER KEEPER
68	PI 142821	CHOGHONDAR	164	NSL 6624	HALF SUGAR ROSE
69	PI 140358	No. 6899	165	NSL 80222	RS-2
70	PI 590697	SP70756-0	166	PI 177269	KOCABAS
71	Ames 8280	B138	167	Ames 2657	SLC 129
72	PI 590616	ELITE DESPREZ TYPE R	168	Ames 2665	U 5201
73	PI 142823	CHOGHONDAR	169	NSL 114616	SP6926-0
74	Ames 14432	Bordo-60	170	PI 613270	A 0051
75	PI 256053	No. 2	171	NSL 34674	REDPACK
76	Ames 2644	CT5mm	172	PI 176421	KOCABAS
77	PI 120706	No. 3238	173	PI 590589	MUNERATI ANNUAL (SL 9470)
78	NSL 86577	72/4-4-2-0	174	PI 174058	No. 7764
79	PI 142818	CHOGHONDAR	175	NSL 141986	CS 42
80	Ames 3049	IDBBNR 4803	176	NSL 195503	EL40 BREEDING LINE 24
81	Ames 8294	B197	177	NSL 28041	B 236
82	PI 142817	CHOGHONDAR	178	Ames 8295	B199
83	PI 141909	-	179	NSL 93280	A76-39
84	PI 117117	No. 299	180	NSL 142025	R and G PIONEER
85	Ames 8279	B131	181	NSL 28020	EARLIDARK
86	PI 611060	Swiss chard	182	PI 590658	FC 702/4
87	PI 140353	No. 6369	183	PI 590765	F1006
88	NSL 176303	YUGO 1	184	PI 610321	056
89	PI 140351	No. 6052	185	PI 172734	KOCABAS
90	PI 124404	PALAG	186	PI 613266	A 0010
91	PI 140362	No. 7249	187	PI 610316	1502aa
92	Ames 8282	B140	188	Seranada	Commercial Variety
93	PI 165062	PAUCAR	189	Varias	Commercial Variety
94	PI 171519	No. 7167	190	Evelina	Commercial Variety
95	PI 164805	CHOGHUNDAR	191	Jaguar	Commercial Variety
96	PI 140350	No. 5926	192	Balaban	Commercial Variety

## Data Availability

All data needed to conduct this study is provided within the manuscript.
